# Evaluating the effectiveness of the safety experience room, an affordable interactive education intervention to prevent unintentional injury among rural preschoolers in China: protocol for a cluster randomized controlled trial

**DOI:** 10.1186/s12889-023-15432-1

**Published:** 2023-03-20

**Authors:** Jieyi He, Wanhui Wang, Peishan Ning, David C. Schwebel, Yang Yang, Peixia Cheng, Jie Li, Min Zhao, Weiqiang Li, Na Zhang, Han Liu, Guoqing Hu

**Affiliations:** 1 Department of Epidemiology and Health Statistics, Hunan Provincial Key Laboratory of Clinical Epidemiology, Xiangya School of Public Health, Central South University, Changsha, Hunan China; 2 Department of Psychology, University of Alabama at Birmingham, Birmingham, AL United States of America; 3 Department of Statistics, Franklin College of Arts and Sciences, University of Georgia, Athens, GA United States of America; 4 Department of Child, Adolescent and Women’s Health, School of Public Health, Capital Medical University, Beijing, China; 5 National Clinical Research Center for Geriatric Disorders, Xiangya Hospital, Central South University, Changsha, Hunan China

**Keywords:** Unintentional injury, Preschooler, Rural area, China, Interactive safety education, Cluster randomized controlled trial

## Abstract

**Background:**

Interactive and experiential learning programs have proven effective to teach children safety and prevent child unintentional injury. However, previously-published programs were designed primarily to address safety concerns of children living in urban, well-resourced areas, and therefore might be less effective or economically infeasible to distribute to children in resource-limited areas, such as those living in rural areas or underdeveloped regions. This proposed study will evaluate the effectiveness of teaching children safety lessons to rural preschoolers in China through the preschool-based Safety Experience Room intervention that was developed based on relevant theories, the lessons of previous intervention research, the characteristics of child injuries in underdeveloped rural areas, and the needs and circumstances of rural families and preschools in China. The study will also evaluate the cost-effectiveness of delivering the program.

**Methods and analysis:**

A single-blinded, 12-month follow-up, parallel-group cluster randomized controlled trial with a 1:1 allocation ratio will be implemented in two selected counties. In total, at least 2378 rural preschoolers aged 3–6 years old will be recruited from 12 preschools, 6 in Yang County and 6 in Shicheng County. Clusters will be randomized at the preschool level and allocated to the control group (routine school-based education) or the intervention group (routine school-based education plus the Safety Experience Room education). External support strategies will be implemented by local partners to minimize attrition. Data collection will be conducted at baseline and then every 3 months during a 12-month follow-up time period. Intention-to-treat (ITT) data analysis will be used. Generalized estimation equations (GEE) will evaluate the effectiveness of the program and generalized cost-effectiveness analysis (GCEA) will evaluate the cost-effectiveness of it. A per-protocol (PP) sensitivity analysis will assess the robustness of ITT results. Subgroup analyses will be performed to evaluate the impact of socio-demographic factors on the intervention effect, following the same strategies as the primary analyses.

**Discussion:**

The newly-designed Safety Experience Room program is expected to be feasible, effective, and financially beneficial. If these hypotheses prove true, we will take steps to disseminate the program to rural preschools across China.

**Trial registration:**

Chinese Clinical Trial Registry (http://www.chictr.org.cn), CHiCTR2000038025, registered on 8 September 2020.

**Supplementary Information:**

The online version contains supplementary material available at 10.1186/s12889-023-15432-1.

## Background

Among the widely-disseminated child safety education topics are school-based lessons to children about how to interact in a safe way with their surroundings. Such efforts can improve children’s safety-related knowledge, skills, behaviors, and practices, and ultimately reduce the occurrence and severity of injury events [1–3]. Traditional school-based education programs where a teacher delivers safety information to children, however, show mixed effectiveness on reducing child injury. They are especially ineffective in transmitting knowledge, skills, and behavior change when lessons lack interactive aspects and are delivered in a monotonous and rote manner [3, 4].

Interactive education represents an educational approach that integrates traditional design, product design, and new media design to enrich the educational experience through communication and interchange among participants that facilitates learning and behavior change [5, 6]. Interactive education can stimulate the improvement of knowledge, attitudes, and behaviors [7] through immersive experiences and participatory learning in diverse forms, such as using miniatures [8], story-based toys or puzzles [9, 10], scenario-based storybooks [11], virtual reality [12], or interactive games [7, 13].

Several published studies report the effectiveness of interactive education interventions to improve children’s knowledge, behaviors, skills, and/or self-efficacy for safety promotion and injury prevention [8, 14–16]. For instance, young children exposed to interactive education programs were found to have improved knowledge, behaviors, skills, or self-efficacy for broad injury prevention [10], as well as specific safety domains like fire safety [17], dog bite prevention [18, 19], and street-crossing [20–23]).

Previous research suffers from at least four limitations. First, existing programs tend to focus on resource-rich children and families. The contents and cost of interventions designed for these populations may not meet the needs of children living in resource-limited areas, including those in rural areas and in impoverished families. Financial barriers are of particular concern when translating existing interventions to underserved children. To be fully effective in resource-limited areas, the cost of implementing an intervention must be feasible and affordable. Second, previous studies generally focus on single injury causes, such as road traffic injuries [8, 22–24] or dog bites [18]), rather than the full array of risky situations and environments that children may face. Third, most previous studies examined changes in injury-related knowledge, attitudes, or simulated behaviors as study outcome measures rather than collecting data on actual injury incidents [25, 26]. This is partly the result of the short durations used to implement interventions with comparatively small sample sizes, making it infeasible to detect significant changes in injury incidence [18, 21–23, 27]. Last, a large portion of the existing studies conducted in low- and middle-income countries (LMICs) rely on pre-post or other non-experimental designs rather than more rigorous randomized controlled trials (RCTs).

To address these research limitations, we developed a standardized, affordable, replicable, and easy-to-implement interactive intervention, the “Safety Experience Room”, to train preschool-aged children in rural China on safety and injury prevention. The program is based on relevant educational theories, extends lessons learned from previous interactive learning intervention research, focuses especially on the characteristics of injuries in rural and resource-limited areas, and considers the specific contextual needs and circumstances of rural preschoolers and preschools in China. We propose a rigorous cluster randomized controlled trial that uses injury incidents as a study outcome to evaluate effectiveness and cost-effectiveness of the Safety Experience Room in preventing unintentional injury incidence among preschoolers living in rural China.

## Methods and analysis

### Study design

A single-blinded, 12-month follow-up, cluster randomized and controlled trial with a 1:1 allocation ratio will be implemented in Yang County, Shaanxi Province, and Shicheng County, Jiangxi Province, China. The trial was registered on September 8, 2020 in the Chinese Clinical Trial Registry (http://www.chictr.org.cn/showproj.aspx?proj=57719, registration number: ChiCTR2000038025).

### Sample size

Based on a previous survey [28], we conservatively estimated that unintentional injury incidence among preschoolers aged 3–6 years old in the study sites over a 12-month period will be 30%. According to relevant reports [29], we estimated the effect size of the Safety Experience Room intervention will be 0.75 (incidence rate ratio) compared with a traditional educational intervention. An intra-class correlation (ICC) of 0.005 within a cluster size of 200 children per preschool was estimated [30, 31]. Nearly whole rural preschoolers stay enrolled in the same preschool throughout childhood, so we conservatively estimate a maximum attrition rate of 10% over the 12-month follow-up time period. Given these estimates, a minimum sample size of 2378 preschoolers is sufficient to achieve statistical power of 80% at the significance level of 0.05. Our preliminary surveys indicate most local rural preschools have 200 to 300 children, so 12 preschools will be adequate to recruit the needed sample size.

### Preschool recruitment

**Fig. 1:**
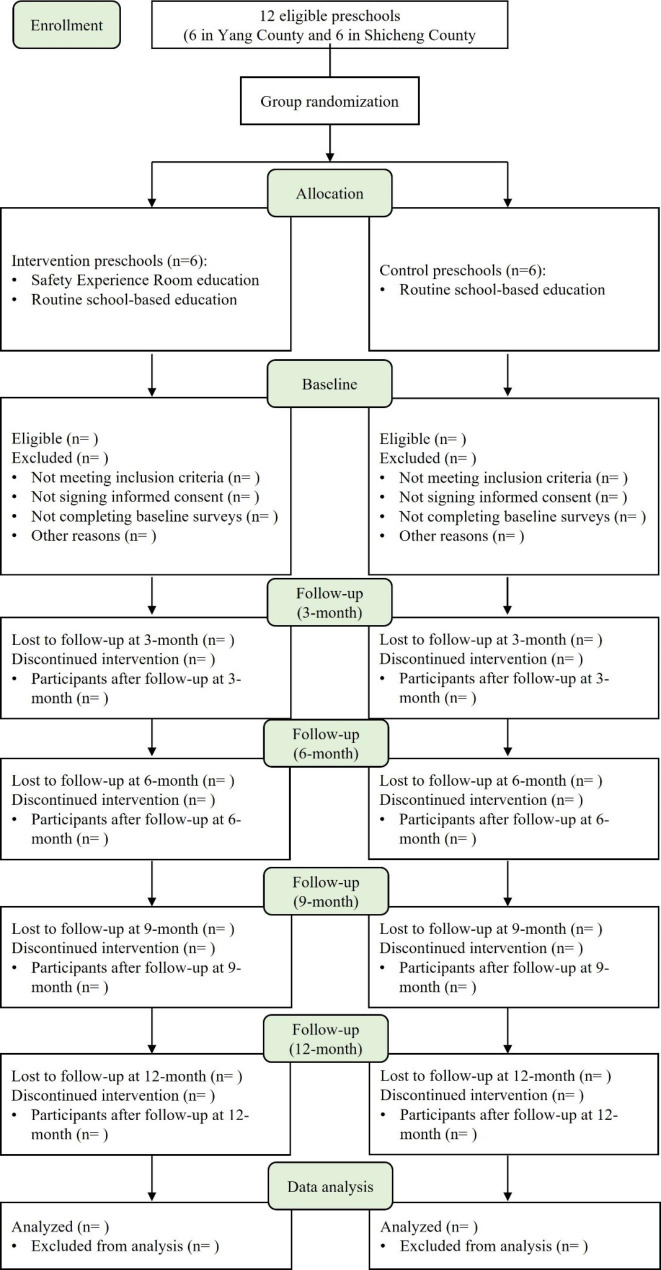
Flow diagram of selection of study participants

**Fig. 2:**
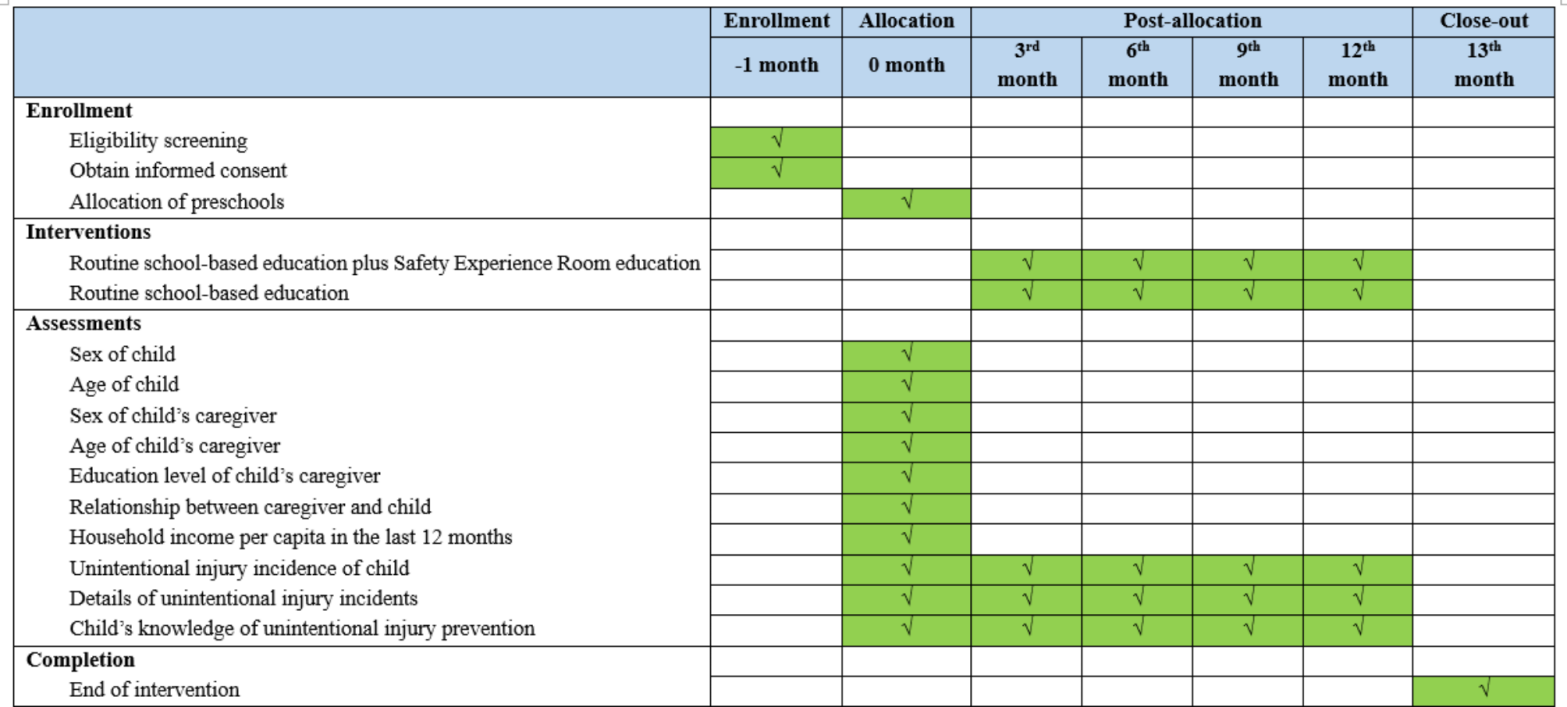
Timeline for participant enrollment, intervention implementation, and assessment

This study plans to recruit study participants from 12 preschools in China, 6 from Yang County in Shaanxi Province, and 6 from Shicheng County in Jiangxi Province (Figs. 1 and 2). Preschools that enroll more than 200 students and agree to participate in this study will be considered eligible preschools.

Each invited preschool will receive an official invitation letter along with relevant project materials. To avoid contamination between children within the same preschool, we will randomize the intervention and control programs at the school level. Consequently, 6 preschools will be randomly allocated to the intervention group, including 3 from Yang County and 3 from Shicheng County, while the other 6 preschools will be allocated to the control group, also including 3 from Yang County and 3 from Shicheng County.

### Participant recruitment

All students attending participating preschools will be eligible to enroll in the study. Exclusion criteria include: (1) children who regularly attend preschools less than 3 full school days a week; (2) preschoolers who have mental or physical disabilities that prohibit valid participation in the learning activities of the Safety Experience Room; (3) preschoolers whose caregivers cannot cooperate with follow-up visits; and (4) preschoolers whose caregivers decline to sign informed consent.

In coordination with local governmental departments and agencies, we will recruit classroom teachers and school staff to help coordinate recruitment of study participants in each selected preschool. The teachers and staff members at each school will initially inform eligible preschoolers’ caregivers about the study via social media platforms (WeChat and QQ, the most popular applications in China), and then organize a caregiver-teacher meeting to communicate further about the study. Caregivers who agree to have their children participate in the study will be provided with an invitation letter that includes the benefits and responsibilities of participating in this study and be invited to read and sign an online informed consent.

Following consent processes, all participating caregivers will complete an online baseline survey to provide information on socio-demographic characteristics and details about unintentional injuries the children experienced in the prior 3 months, including the type and severity of each injury.

### Randomization and masking

Once recruited, each enrolled preschool will be randomly allocated to a group by an independent (masked) researcher. Block randomization with a block size of 2 will be used to ensure balance between intervention and control groups. The allocation sequence will be generated by R version 4.0.4 software and the sequence will be sequentially numbered and opaque, stored in sealed envelopes until interventions are assigned. Group allocation will be concealed during data analysis.

### Interventions

Both the intervention and control groups will receive routine school-based education on safety and injury prevention from their preschool. This education generally encompasses general health knowledge and skills, including injury prevention, and is performed by preschool teachers.

The intervention group will also receive the Safety Experience Room intervention. The Safety Experience Room involves creation of a space designed to teach safety and injury prevention. It can easily be renovated from typical Chinese preschool classrooms according to the standards in Table 1.

**Table 1:** General description of the Safety Experience Room intervention design

Item	Requirement
**I. Infrastructure**	
1. Space	A room with at least 60 square meters
2. Furnishings	(1) 65-inch multimedia computer with touch screen (2) Moveable computer holder (3) Bookshelves (4) Child-sized chairs (5) Office chair and table for instructor (6) Teaching props to simulate traffic environments, including models of pedestrian traffic light, zebra crossing, traffic signs, and vehicles (7) Teaching props to simulate natural water environments, with models of inflatable ball pool (simulated water area), fences, safety warning signs, and lifesaving equipment
3. Accessories	Floor covered by soft mats
4. Functional zones	(1) Multi-media zone (2) Simulated scenario zone (3) Spare zone
**II. General description and contents of the Safety Experience Room intervention**	
1. Target population	Preschoolers
2. Limit of participants	No more than 30 preschoolers at a time
3. Instructor	Preschool teachers or staff who receive standardized training
4. Guidance material	Operation Manual of the Safety Experience Room
5. Safety themes	
(a) Road traffic injury	(1) Learn basics of pedestrian traffic lights and zebra crossings – their appearances, functions, and purpose of usage (2) Never play around parked or moving vehicles or near roadways
(b) Falls	(3) Walk up and down stairs cautiously and do not climb on or near high indoor places such as tables, shelves, closets, cabinets, windows, or balconies (4) Never stand in puddles or on rocks, and do not climb high places outdoors without protection, such as trees, rocks, fences, or walls
(c) Drowning	(5) Seek adults for help when a person falls into the water (6) Swim only in places permitted by the government, with full-time adult supervision, and while wearing safety floats
6. Instructional materials	(1) Animation videos (2) Interactive games (teaching version) (3) Teaching props for simulations (4) Series of large-sized story books and the corresponding story recordings
7. Teaching activities	(1) Watching animation videos (2) Playing interactive games (teaching version) (3) Experiencing simulations of actual traffic and natural water environments (4) Reading story books and listening to story recordings (5) Summarizing key safety behaviors of the learning courses
8. Number of classes	Six 40-minute lessons, one weekly for six weeks. Each lesson has a single theme. Complete lessons are required to be implemented twice in a single semester.
**III. Learning evaluation**	
1. Evaluation tool	Evaluation version of the interactive games
2. Guidance material	Operation Manual of the Safety Experience Room

**Fig. 3:**
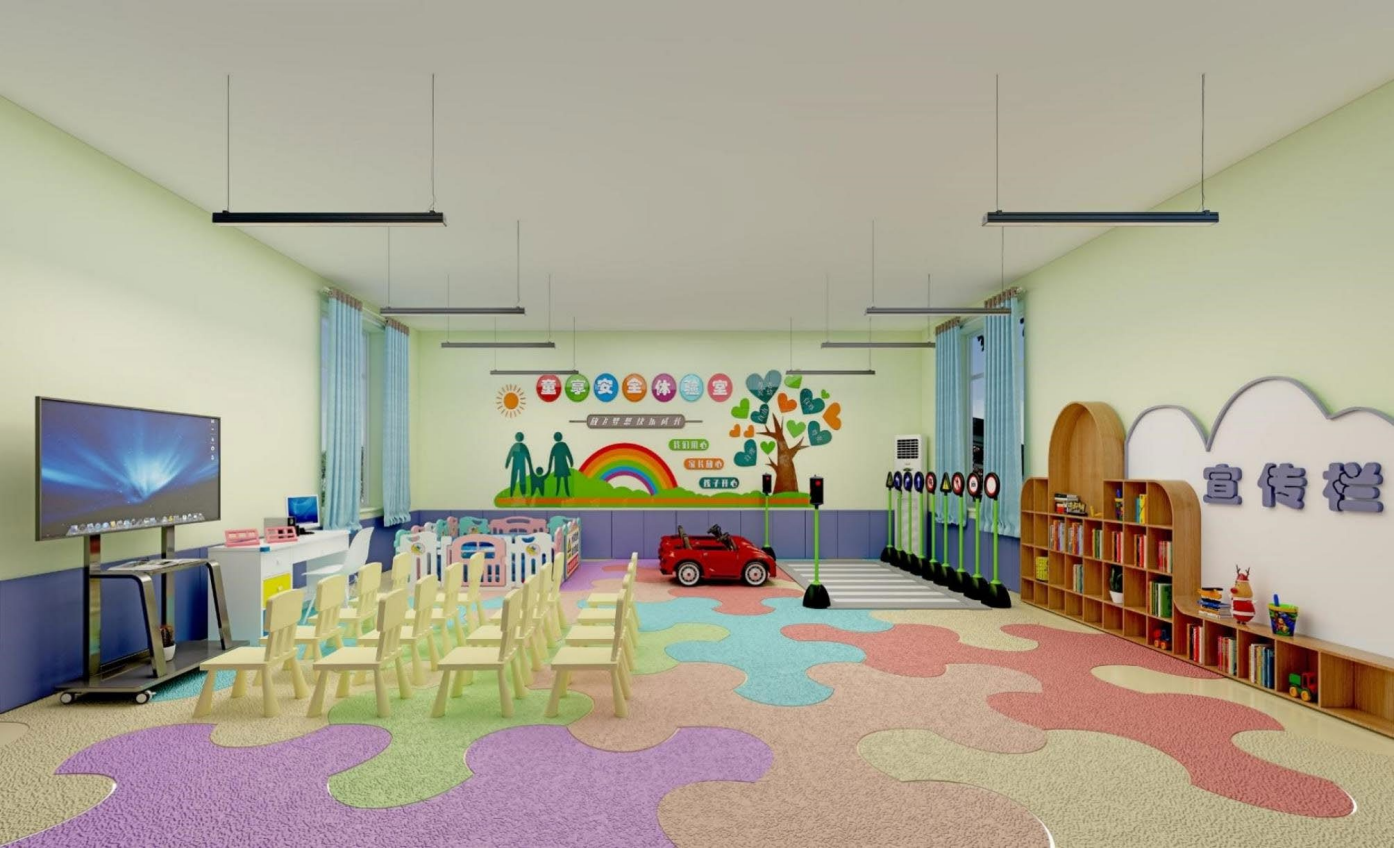
Example interior design of the Safety Experience Room in a preschool

The Safety Experience Room will be flexibly divided into 3 zones, each with a specific function: a multi-media zone, a simulated scenario zone, and a spare zone (Fig. 3). The total cost of furnishing and decorating each Safety Experience Room will be controlled to a maximum cost of 13,200 CNY (approximately 1,970 USD), as detailed in Table 2.

**Table 2:** Budget to developing a typical Safety Experience Room

Item	Unit price ($)	Number	Amount ($)
65-inch multimedia touchscreen computer	$776	1	$776
Moveable computer holder	$102	1	$102
Bookshelves	$81	1	$81
Child-sized chairs	$4	30	$120
Office chair and table for instructor	$58	1	$58
Teaching props for simulations	$597	1	$597
Floor mats	$233	1	$233
**Total**			**$1967**

Note: Unit price and amount are presented in U.S. dollars and estimated based on the exchange rate of 6.70 Chinese Yuan to 1 U.S. dollar

Consistent with stages of preschooler’s cognitive development [32, 33], we developed 3 sets of informative and entertaining instructional materials originally to teach children about each of the 6 safety themes. The lessons are designed to be engaging and easy-to-learn and are delivered through animation videos, interactive games with coherent game plots, and large-sized picture story books with corresponding story recordings. All teaching and learning materials were developed based on three overriding principles: (a) content to address risky scenarios that rural Chinese children commonly face in daily life and information to help preschoolers react safely in these situations; (b) content that teaches preschoolers to identify dangerous behaviors and recognize the potential for serious injurious consequences from the dangerous behaviors; and (c) a summarization of essential safety rules that children should obey and reminder to avoid dangerous behaviors at the end of each instructional material.

**Fig. 4:**
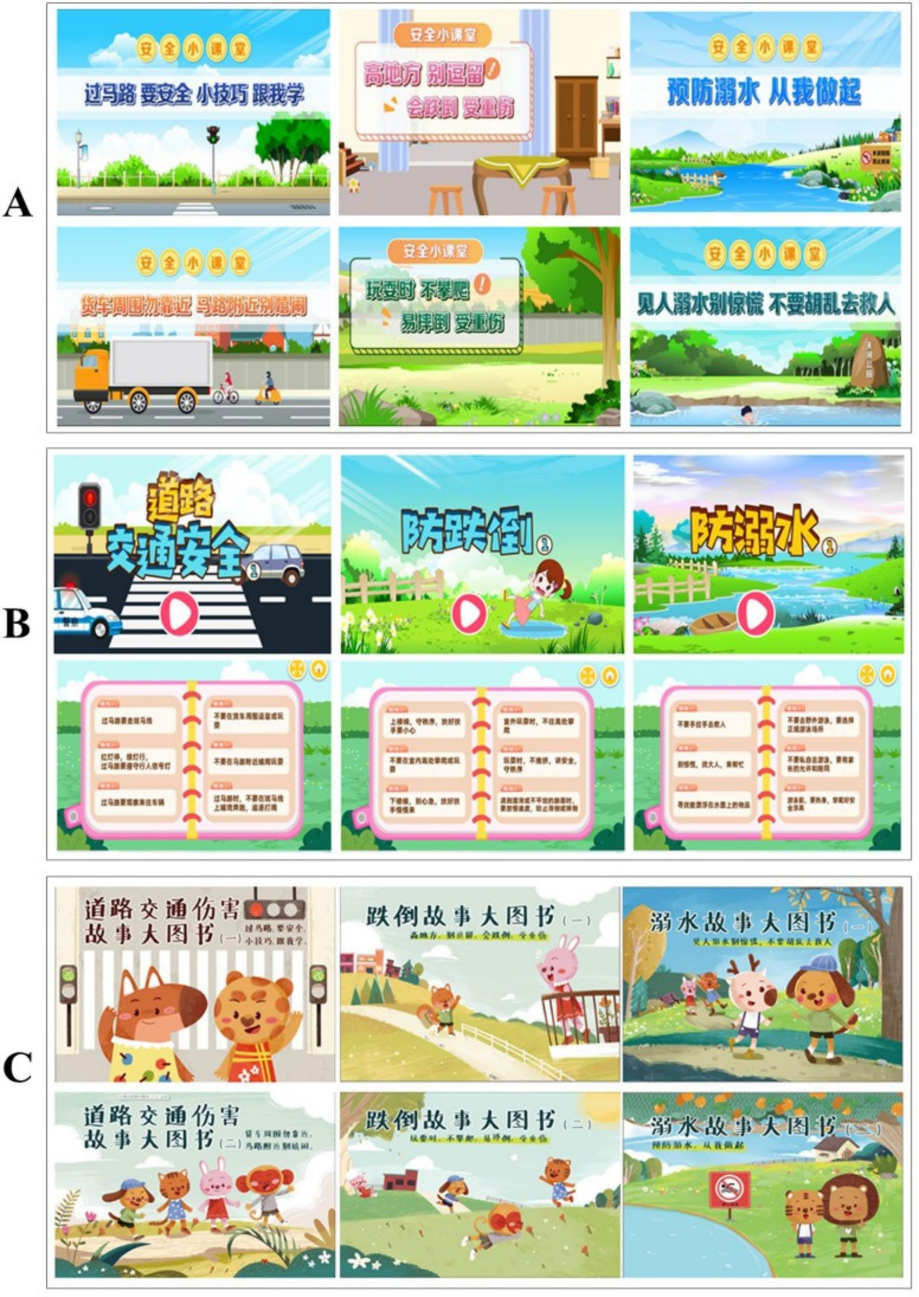
Instructional materials of the Safety Experience Room intervention (A. animation videos; B. interactive games; C. large-picture story books)

All content was developed properly with health behavior change theory in mind. In particular, the content had goals to alter peer norms by presenting safe behavior as normative; to instill in children a need to follow safety rules; to teach children about the risk and potential severity of injury at a developmentally-appropriate level; and to create some developmentally-appropriate levels of fear of injury in children. Some content is delivered using animals as characters to improve race, ethnicity, cultural, and gender identification among the children viewing it. The content will be delivered concretely through following primary mechanisms:

1. animation videos (Fig. 4A), which disseminate unintentional injury prevention knowledge that children should have in daily life through the activities of lively and entertaining cartoon characters;
2. interactive games (Fig. 4B), which are delivered by the multimedia touchscreen computer in the Safety Experience Room to offer children choice-points that involve safe or risky decisions and illustrate the health outcomes of distinct decisions. As children progress through the discrepant game plots, they become familiar with the consequences of potentially injurious decisions, both through the game plots and through reinforcing instructions from their teachers;
3. large-sized picture books and story recordings (Fig. 4C), which convey unintentional injury prevention knowledge and information through animal cartoon characters and voice-over story recordings.

For the two teaching themes of road traffic injury and drowning, we will utilize teaching props that simulate traffic and natural water environments in the simulated scenario zone. These lessons will convey relevant safety behaviors to children through learning tasks guided by their instructors.

To summarize, the Safety Experience Room offers five important educational aspects to impart safety under the guidance of the instructor (Table 1):

1. watching animation videos on a multimedia computer with touchscreen;
2. playing interactive games with coherent game plots using the large touchscreen multimedia computer;
3. experiencing scenarios in simulated traffic and natural water environments;
4. reading large-sized picture story books and listening to relevant story recordings related to the learning themes;
5. hearing summaries of the key safety behaviors from the teachers at the end of each course.

Instruction will be conducted over a six-week period, with one theme conveyed each week. To enhance learning among this young group of children, we will expose the children to a similar set of lessons a second time, over the next six weeks. Repetition of lessons should enhance learning and memory, which we expect to translate to safer behaviors outside the classroom [20].

If the Safety Experience Room intervention proves feasible and effective as hypothesized, we will deliver the intervention to children in the control group after completing the entire intervention, and then disseminate it broadly to other rural preschoolers across China.

### Intervention implementation

An operation manual was developed to ensure standardized implementation of the Safety Experience Room intervention. The manual consists of eleven chapters which together introduce the design, facilities, and class schedule for the Safety Experience Room intervention; elaborate the lessons that teachers deliver; specify strategies to evaluate the intervention’s effectiveness using the evaluation version of the interactive games; and inform the management and maintenance staff at the facilities about the teaching materials in the Safety Experience Room.

Along with distributing the operation manual, we will organize a standardized training session for all instructors involved in the intervention group to improve compliance with the operation manual and ensure all instructors implement the Safety Experience Room education and evaluation program in a standard way.

### Feasibility testing

Before the formal study begins, preschool teachers involved in the intervention group will undertake feasibility testing. The teachers will conduct pilot teaching sessions with all lessons in one class to familiarize themselves with the Safety Experience Room intervention and master the lesson delivery. Qualitative data will be collected from teachers and used to refine the teaching materials and teaching practices.

### Fidelity of data collection

With the support from local partners including the local education bureau, the local office of the charitable foundation “World Vision,” and the preschools participating in this study, a coordinating group will be established for each enrolled preschool to assist the preschool teachers and staff in consistent delivery of the Safety Experience Room intervention in a manner consistent with the operation manual and to collect the survey data from preschoolers and their caregivers.

## Outcome measures

### Primary outcome measure

The primary outcome measure will be the unintentional injury incidence rate among preschoolers over the 12-month study period, which will be calculated by combining data from the four follow-up evaluations. Follow-up evaluations will be conducted quarterly to minimize caregiver’s recall bias for minor or moderate injuries experienced by the child [34].

We define an injury event as an incident meeting any of three criteria: (1) child receives a medical diagnosis or treatment by a doctor or other medical professional following an injury; (2) child receives first aid, takes any injury-related medication, or receives massage or cold/hot compress by a family member, preschool teacher, preschool staff member, or other individuals following an injury; or (3) child is restricted from school or other activities, or is forced to stay in bed or rest for more than a half-day following an injury. If a caregiver reports that a child experienced more than one unintentional injury event in the prior 3 months, we will record the number of injury events and then collect specific information regarding the most severe one.

The unintentional injury incidence rate will be calculated as the “number of preschoolers who experience new unintentional injury events over the past year divided by the total number of preschoolers×100%”.

### Secondary outcome measures

We will collect data concerning several secondary outcome measures, as detailed below.

1. Preschooler’s unintentional injury prevention knowledge for road traffic injury, falls, and drowning. This knowledge will be evaluated based on the learning content of the 6 educational themes children are exposed to in the Safety Experience Room. Table 3 lists 18 key items we will use to measure children’s unintentional injury prevention knowledge.
2. Cost-effectiveness analysis (CEA). The calculation of CEA will follow the World Health Organization CHOosing Interventions that are Cost-Effective (WHO-CHOICE) approach to estimate population level effects of the interventions using a form of generalized cost-effectiveness analysis (GCEA) [35, 36].

**Table 3:** Key unintentional injury prevention behaviors that preschoolers will learn for road traffic injury, falls, and drowning prevention

**1. Road traffic injury**
a. Pedestrians should use zebra crossings to cross the road
b. Pedestrians should only cross the road when they see a green light
c. Even when accompanied by adults to cross the road, children should look left and right
d. Do not stand or play near parked or moving cars or trucks
e. Do not play near the road
f. Do not run, chase, skip, or jump when crossing the road
**2. Falls**
a. Be orderly and hold the handrail carefully when going up stairs
b. Do not climb or play at high places, such as tables, shelves, closets, cabinets, windows, and balconies
c. Hold the handrail carefully and do not jump, leap, or hurry down stairs. Also, do not crowd others.
d. Do not climb high trees, rocks, fences, or walls when playing outdoors
e. Do not push or shove other children when playing outdoors
f. Walk or run with caution, and do not stand in puddles or on rocks
**3. Drowning**
a. Never attempt to save a child who falls into the water by yourself or with other children
b. Seek adults for help when a person falls into the water
c. Learn what common objects can float on the water and might act as water rescue instruments
d. Never swim in or play around natural water by yourself or with other children but no supervising adults
e. Never swim without adult supervision
f. Wear safety floats while swimming if you do not know how to swim

For this study, the health effect of the Safety Experience Room intervention is the difference in the number of preschoolers experiencing unintentional injury events over the 12-month intervention period between the intervention and control groups. The costing of the intervention will include subsidies to the instructors paid from the project and costs for designing, furnishing and decorating each Safety Experience Room. The cost effectiveness ratio will be calculated as the total implementation costs of the intervention group divided by the health effect. Monte Carlo simulation (1000 runs using a truncated normal distribution) will be used to calculate a 95% uncertainty interval of the cost and outcome data.

### Formal data collection

Survey data regarding the primary outcome for both groups, injury incidents, will be collected at baseline and every 3 months throughout the 12-month follow-up time period. In coordination with the local education bureau, the preschools will organize a caregiver-teacher meeting every 3 months. The class teacher will send the online survey link and ask all caregivers to complete the survey at the caregiver-teacher meeting. All primary outcome data will be collected through online survey. If caregivers fail to complete the online questionnaire, the classroom teacher will contact them to collect the survey data.

Collection of secondary outcome data will be carried out at baseline for both groups and after the intervention group completes each safety-related theme course designed for the intervention group. Secondary outcome data will be collected simultaneously from the control group to maintain comparison between two groups, but we will not disseminate any correct answers or teachings to the children in the control group before the study ends to avoid contamination in that group. All secondary outcomes data will be collected via preschoolers playing an evaluation version of the interactive games that has different story plots from the teaching version and permits retention of evaluation results. The evaluation version of the interactive games will not include any modules that disseminate injury prevention information or display evaluation results to children. Administration of the assessment will occur in a quiet environment. Each child will complete the tasks individually in the Safety Experience Room, with other classmates staying in the classroom respectively during the evaluation.

In order to assess children in the control group, relevant hardware and software (the multimedia touchscreen computer with the evaluation version of interactive games installed) will be delivered to control preschools accordingly. Staff members and teachers will help children start and reset the interactive games as well as maintain a quiet evaluation environment during the evaluation process.

Before formal data collection, teachers involved in both groups will learn to operate the evaluation games. Teachers will enter each child’s ID number and supervise individualized data collection but will not communicate with children or see children’s evaluation results. All data will be stored in the game backend database.

### Data analysis plan

Primary data analysis will employ an intention-to-treat (ITT) approach. Descriptive statistics will be calculated for socio-demographic variables and primary and secondary outcomes, including mean (or median) and standard deviation (or range and interquartile range) for continuous variables, and frequency and proportion for categorical variables. At each time point, Chi-square test and two-sample t-test (or Wilcoxon rank sum test if data are skewed) will be used to examine the differences in outcome measures between intervention and control groups for categorical and continuous variables, respectively. Trend tests or analysis of variance (ANOVA) will be used to detect within-group differences across the four follow-up visits.

We will also use Generalized Estimating Equation (GEE) and Generalized Linear Mixed Models (GLMM) to evaluate the overall effectiveness of the intervention after adjusting for socio-demographic variables, engagement, and baseline injury events. Both binary outcomes (whether experienced unintentional injury during the study period) and quantitative count outcomes (number of unintentional injury events and injury prevention knowledge score) at the individual level will be analyzed using these models. Zero-inflation models will be used when needed.

Missing values will be imputed using either the Expectation Maximization (EM) algorithm or Markov Chain Monte Carlo, as appropriate for the data. A per-protocol (PP) sensitivity analysis will be conducted to validate ITT results. All statistical analyses will be performed using the up-to-date version of R. All statistical tests will be two-sided, with the level of 0.05 considered statistically significant.

Subgroup analyses will be performed to assess the impact of socio-demographic factors on the intervention effectiveness, including sex and age of children, grade level of preschoolers, education level of preschooler caregivers, and household income per capita in the prior 12 months. Subgroup analyses will follow the same statistical strategies for primary analyses.

This study will be rigorously conducted, analyzed, and reported in accordance with the Standard Protocol Items: Recommendations for Interventional Trials (SPIRIT) 2013 statement [37] and the Consolidated Standards of Reporting Trials (CONSORT) 2010 statement [38].

## Discussion

Interactive education has become increasingly popular worldwide given widespread availability of intelligent and multi-media devices at reasonable cost, plus the capacity to ground interactive interventions in educational and developmental theory and their proven efficacy [7, 39]. However, high-quality research evidence targeting child injury outcomes remains absent for interactive education interventions designed to prevent child unintentional injury in resource-limited areas like rural China.

We developed a standardized interactive education intervention that is easily replicated and generalized, the Safety Experience Room, and designed a 12-month RCT to assess the program’s effectiveness and cost-effectiveness in reducing child unintentional injury incidence rate in rural China. The intervention design relies on relevant theories, the lessons of previous intervention research, the characteristics of injuries in rural settings, and the cultural context of rural preschoolers and preschools in China.

This study will adhere strictly to recommended guidelines for design and implementation of randomized controlled trials [37, 38]. High-quality evidence will be generated to determine the effectiveness and cost-effectiveness of the Safety Experience Room intervention, and expand existing evidence on the effectiveness of interactive education interventions to prevent child unintentional injury in resource-limited areas.

If the Safety Experience Room education intervention proves effective as well as economically and technically feasible as we hypothesize, we will move to disseminate the standard, affordable, and easy-to-implement program broadly in rural China [7, 8, 40].

## Electronic supplementary material

Below is the link to the electronic supplementary material.

Supplementary Material 1

## Data Availability

Data sharing is not applicable to this article as no datasets were generated or analysed during the current study.

## List of Abbreviations

ANOVA: Analysis of variance
CEA: Cost-effectiveness analysis
CONSORT: Consolidated standards of reporting trials
EM: Expectation maximization
GCEA: Generalized cost-effectiveness analysis
GEE: Generalized estimation equations
GLMM: Generalized linear mixed model
ICC: Intra-class correlation
ITT: Intention-to-treat
LMICs: Low- and middle-income countries
PP: Per-protocol
RCT: Randomized controlled trial
SPIRIT: Standard protocol items, recommendations for interventional trials
WHO-CHOICE: World Health Organization choosing interventions that are cost-effective
